# Preference reversal in quantum decision theory

**DOI:** 10.3389/fpsyg.2015.01538

**Published:** 2015-10-08

**Authors:** Vyacheslav I. Yukalov, Didier Sornette

**Affiliations:** ^1^Department of Management, Technology and Economics, ETH ZürichZürich, Switzerland; ^2^Bogolubov Laboratory of Theoretical Physics, Joint Institute for Nuclear ResearchDubna, Russia; ^3^Swiss Finance Institute, University of GenevaGeneva, Switzerland

**Keywords:** preference reversal, decision theory, uncertainty, behavioral quantum probability, planning paradox

## Abstract

We consider the psychological effect of preference reversal and show that it finds a natural explanation in the frame of quantum decision theory. When people choose between lotteries with non-negative payoffs, they prefer a more certain lottery because of uncertainty aversion. But when people evaluate lottery prices, e.g., for selling to others the right to play them, they do this more rationally, being less subject to behavioral biases. This difference can be explained by the presence of the attraction factors entering the expression of quantum probabilities. Only the existence of attraction factors can explain why, considering two lotteries with close utility factors, a decision maker prefers one of them when choosing, but evaluates higher the other one when pricing. We derive a general quantitative criterion for the preference reversal to occur that relates the utilities of the two lotteries to the attraction factors under choosing vs. pricing and test successfully its application on experiments by Tversky et al. We also show that the planning paradox can be treated as a kind of preference reversal.

## 1. Introduction

For many decades, psychologists and economists have been intrigued by a seemingly anomalous effect termed *preference reversal*. The simplest example illustrating this effect is as follows. First, subjects are asked to choose between two lotteries, say *L*_1_ and *L*_2_, such that *L*_1_ has a high chance to win a relatively modest prize, while *L*_2_ offers a lower chance of winning, but an essentially larger prize. The majority of subjects choose the more certain win of lottery *L*_1_, despite the fact that lottery *L*_2_ can enjoy a larger expected utility. Then subjects are asked to price each of the lotteries, as if they would own them and wish to sell the right to play them. Surprisingly, the majority of subjects price higher the less certain lottery *L*_2_ in apparent contradiction with their previous choice. This example embodies the essence of the preference reversal effect.

Among the first scientists emphasizing the existence of this effect were Lindman (1971) and Lichtenstein and Slovic (1971, 1973). Their studies were followed by several authors demonstrating the occurrence of this effect in psychology and economics (Grether and Plott, 1979; Loomes and Sugden, 1983; Holt, 1986; Goldstein and Einborn, 1987; Karni and Safra, 1987; Segal, 1988; Tversky et al., 1988; Schkade and Johnson, 1989). Many other citations can be found in the review articles (Slovic and Lichtenstein, 1983; Tversky and Thaler, 1990; Tversky et al., 1990). The experimental studies have established the clear validity and robustness of the preference reversal phenomenon.

The preference reversal effect looks surprising because, according to the common understanding of utility, the choice among the given lotteries should be based on the objective values of the latter, thus, being procedure invariant. Since the lottery values are not changed, why then is the preference reversed?

It has been proved by Tversky and Thaler (1990) and Tversky et al. (1990) that it is the *breaking of procedure invariance* that is responsible for the preference reversal phenomenon. It turns out that subjects weight more heavily payoffs in pricing than in choice, so that the preference reversal is a purely psychological effect.

The origin of the preference reversal has been recently explained from the point of view of neurology by Kim et al. (2012). It has been experimentally shown that there exists correlation between visual fixation and preferences. Visual fixations both reflect and influence preferences. From one side, these fixations reflect which objects seem to be more important for the subject. And, from the other side, such fixations modulate the neural correlates of preferences, with activity in ventromedial prefrontal cortex and ventral striatum, reflecting the value of the fixated item compared to the value of the item not fixated. Kim et al. studied the process of decision making under risk and measured eye movements while people chose between gambles or bid in pricing gambles. Consistently with the previous work, they found that, for two gambles matched in expected value, people systematically chose the higher probability option, but requested a higher ask price for the option that offered the greater amount to win, thus demonstrating preference reversal.

This effect was accompanied by a shift in fixation of the two attributes, with people fixating more on probabilities during choices and more on amounts during selling. In this way, there exists *probability-vs.-amount dichotomy*: When choosing, one pays more attention to probabilities while, when selling, one better appreciates amounts.

Understanding the cause of the preference reversal is the first necessary step. The next step should be the description of this effect by a mathematical model. Previous suggested models were not successful, as was analyzed by Tversky and Thaler (1990) and Tversky et al. (1990). In the present paper, we show that the effect of preference reversal finds a simple and natural explanation in the frame of the Quantum Decision Theory developed by the authors (Yukalov and Sornette, 2008, 2009a,b, 2010, 2011, 2013, 2014a,b, 2015).

## 2. Basics of quantum decision theory

There exists several approaches applying quantum notions to psychological sciences, as can be inferred from the books (Khrennikov, 2010; Busemeyer and Bruza, 2012; Bagarello, 2013; Haven and Khrennikov, 2013) and the review articles (Yukalov and Sornette, 2009b; Busemeyer et al., 2014; Sornette, 2014; Ashtiani and Azgomi, 2015), where numerous citations to the previous literature can be found. Quantum Decision Theory (QDT) principally differs from all those approaches in two aspects. First, QDT is based on a self-consistent mathematical foundation that is common for both quantum measurement theory and quantum decision theory. Starting from the von Neumann (1955) theory of quantum measurements, we have generalized it to the case of uncertain or inconclusive events, making it possible to characterize uncertain measurements and uncertain prospects. Second, the main formulas of QDT are derived from general principles, giving the possibility of quantitative predictions, without fitting parameters. This is in contrast with the usual way of constructing particular models for describing some concrete experiments, with fitting the model parameters from empirical data.

We shall not repeat here the mathematical foundation of QDT that has been thoroughly expounded in our previous papers, but we will just briefly recall the resulting formulas that are necessary for describing the preference reversal effect.

Let us consider a composite event, called prospect,

(1) $$\begin{array}{l} \pi_{n}=A_{n}⊗B\;. \end{array}$$

Here *A*_*n*_ is an operationally testable event, represented in a Hilbert space by an eigenstate |*n*〉. While *B* = {*B*_α_, *b*_α_} is an inconclusive event that is a set of possible events *B*_α_, represented in a Hilbert space by eigenstates |α〉, and equipped with random amplitudes *b*_α_, so that the inconclusive event is represented by a state $|B\rangle =\sum_{\alpha }b_{\alpha }|\alpha \rangle$.

The prospect operator is $\hat{P}(\pi_{n})=|nB\rangle \langle nB|$, such that the prospect probability is given by the quantum formula

(2) $$\begin{array}{l} p\left(\pi_{n}\right)=\text{Tr}\hat{\rho }\hat{P}\left(\pi_{n}\right)\;, \end{array}$$

where $\hat{\rho }$ is a strategic state of a decision maker. By construction, the prospect probability enjoys the properties of a probability measure:

(3) $$\begin{array}{l} \underset{n}{\sum }p\left(\pi_{n}\right)=1\;,\;0\leq p\left(\pi_{n}\right)\leq 1\;. \end{array}$$

It is easy to show that the prospect probability takes the form

(4) $$\begin{array}{l} p\left(\pi_{n}\right)=f\left(\pi_{n}\right)+q\left(\pi_{n}\right)\;, \end{array}$$

where the first term is called *utility factor*, characterizing the utility of the prospect, while the second term is *attraction factor* representing behavioral biases.

The intuitive explanation of the above probability expression (4) is straightforward: The definition of a quantum probability (2) for a composite event can be separated into a term containing diagonal matrix elements and a term including off-diagonal elements. The diagonal elements compose the term *f*(π_*n*_), while the off-diagonal elements define the term *q*(π_*n*_). The occurrence of an off-diagonal term is a typical feature of quantum theory, where this quantity is called *interference term* or *coherence term*. The existence of such an interference term constitutes the principal difference of the quantum approach from the classical consideration, where there are no interference terms. It is the appearance of interference terms that makes the structure of quantum expressions richer then the related classical ones and that allows one to explain those psychological phenomena that, otherwise, are inexplicable in classical decision making. Sometimes, the quantum approach even yields conclusions that are impossible in classical decision making, as, for instance, the possibility to agree on disagree (Khrennikov and Basieva, 2014). Below we show that this interference term, composing the attraction factor, is essential in explaining the existence of the preference reversal effect that cannot be described in classical decision theory.

The prospect probability satisfies the quantum-classical correspondence principle.

(5) $$\begin{array}{l} p\left(\pi_{n}\right)\to f\left(\pi_{n}\right)\;,\;q\left(\pi_{n}\right)\to 0\;. \end{array}$$

This defines the utility factor as a classical-type probability, with the standard properties

(6) $$\begin{array}{l} \underset{n}{\sum }f\left(\pi_{n}\right)=1\;,\;0\leq f\left(\pi_{n}\right)\leq 1\;. \end{array}$$

This is equivalent to the normalization condition

$$\begin{array}{l} \underset{n\alpha }{\sum }|b_{\alpha }{|}^{2}\langle n\alpha \;|\;\hat{\rho }\;|\;n\alpha \rangle =1\;, \end{array}$$

imposing a constraint on the random quantities *b*_α_.

When considering lotteries, an event *A*_*n*_ ≡ *A*(*L*_*n*_) implies the choice of a lottery *L*_*n*_. Then the inconclusive set *B* characterizes the decision maker hesitations between uncertain events *B*_α_, describing uncertainty with respect to the decision maker ability and with respect to the lottery formulation (Yukalov and Sornette, 2014b, 2015). The explicit form of the utility factor is given by minimizing the Kullback-Leibler information functional, which in the simple case of uncertainty yields

(7) $$\begin{array}{l} f\left(\pi_{n}\right)=\frac{U\left(L_{n}\right)}{\sum_{n}U\left(L_{n}\right)}\;, \end{array}$$

with *U*(*L*_*n*_) being the expected utility of a lottery *L*_*n*_. Note that the minimization of the information functional results in expression (7) that might be familiar to psychologists as a Luce (1959) choice rule using utility as response strength.

The attraction factor reflects the effects of quantum coherence and interference, and in decision theory it represents the behavioral biases rendering the prospects more or less attractive from the subconscious point of view of decision maker. By their definition, attraction factors lie in the interval

(8) $$\begin{array}{l} -1\leq q\left(\pi_{n}\right)\leq 1 \end{array}$$

and satisfy the *alternation property*

(9) $$\begin{array}{l} \underset{n}{\sum }q\left(\pi_{n}\right)=0\;. \end{array}$$

Also, in the case of non-informative priors, the attraction factors for the considered prospect lattice {π_*n*_ : *n* = 1, 2, …, *N*} obey the *quarter law*

(10) $$\begin{array}{l} \frac{1}{N}\overset{N}{\underset{n=1}{\sum }}|q\left(\pi_{n}\right)|=\frac{1}{4}\;. \end{array}$$

This law makes it admissible to estimate the attraction factors by the values ±0.25, thus quantitatively predicting preferences.

The prospect lattice is ordered by the values of prospect probabilities. A prospect π_*i*_ is termed preferable to π_*j*_ if and only if

$$\begin{array}{l} p\left(\pi_{i}\right)>p\left(\pi_{j}\right)\;\left(\pi_{i}>\pi_{j}\right)\;. \end{array}$$

At the same time, a prospect π_*i*_ is more useful than π_*j*_ when *f*(π_*i*_) > *f*(π_*j*_). A prospect π_*i*_ is more attractive than π_*j*_, when *q*(π_*i*_) > *q*(π_*j*_). In this way, a prospect can be more useful but less attractive, as a result being less preferable.

A necessary condition for the existence of a nonzero attraction factor is that the composite prospect be entangled (Yukalov and Sornette, 2014a, 2015). Otherwise, there is no need of involving quantum probabilities.

## 3. General criterion of preference reversal

Preference reversal may naturally arise in the frame of quantum decision theory. In this section, we derive the general criterion for the occurrence of this effect.

Suppose a decision maker considers a lattice of just two prospects

(11) $$\begin{array}{l} \pi_{n}=A\left(L_{n}\right)⊗B\;\left(n=1,2\right)\;, \end{array}$$

with the intention of choosing between them. Here *A*(*L*_*n*_) implies the action of choosing a lottery *L*_*n*_. And *B* is a set incorporating uncertainties associated with this choice. Let one prefer the prospect π_1_ against π_2_, which means that

(12) $$\begin{array}{l} p\left(\pi_{1}\right)>p\left(\pi_{2}\right)\;\left(\pi_{1}>\pi_{2}\right)\;. \end{array}$$

Taking into account the alternation property, we have

(13) $$\begin{array}{l} q\left(\pi_{1}\right)+q\left(\pi_{2}\right)=0\;. \end{array}$$

This tells us that the prospect π_1_ is preferred to π_2_ if and only if

(14) $$\begin{array}{l} f\left(\pi_{2}\right)-f\left(\pi_{1}\right)<2q\left(\pi_{1}\right)\;. \end{array}$$

Now, assume that the decision maker plans to price the given lotteries, e.g., wishing to sell them. The lotteries remain the same as before. However, uncertainties in selling are of course different from those when choosing, hence, the uncertain set *B*′, associated with selling, is different from the set *B* including uncertainties associated with choosing. Now, the decision maker evaluates the two different prospects

(15) $$\begin{array}{l} \pi_{n}=A\left(L_{n}\right)⊗B^{\prime }\;\left(n=3,4\right)\;, \end{array}$$

where *L*_1_ = *L*_3_ and *L*_2_ = *L*_4_.

Preference reversal implies that, contrary to the situation with choosing, now the decision maker evaluates higher the prospect π_4_ compared to π_3_, so that

(16) $$\begin{array}{l} p\left(\pi_{3}\right)<p\left(\pi_{4}\right)\;\left(\pi_{3}<\pi_{4}\right)\;. \end{array}$$

In view of the alternation property

(17) $$\begin{array}{l} q\left(\pi_{3}\right)+q\left(\pi_{4}\right)=0\;, \end{array}$$

the preference of π_4_ occurs only when

(18) $$\begin{array}{l} f\left(\pi_{4}\right)-f\left(\pi_{3}\right)<2q\left(\pi_{3}\right)\;. \end{array}$$

Since the lotteries are the same (*L*_1_ = *L*_3_ and *L*_2_ = *L*_4_), their expected utilities are pairwise equal: *U*(*L*_1_) = *U*(*L*_3_) and *U*(*L*_2_) = *U*(*L*_4_). Therefore, the utility factors are also pairwise equal

(19) $$\begin{array}{l} f\left(\pi_{1}\right)=f\left(\pi_{3}\right)\;,\;f\left(\pi_{2}\right)=f\left(\pi_{4}\right)\;. \end{array}$$

Combining the above conditions, we obtain the *preference reversal criterion*:

(20) $$\begin{array}{l} 2q\left(\pi_{3}\right)\;<\;f\left(\pi_{2}\right)-f\left(\pi_{1}\right)\;<\;2q\left(\pi_{1}\right)\;. \end{array}$$

Let us stress that in classical decision making, where *q*(π_1_) = *q*(π_3_) ≡ 0, the inequalities (20) cannot hold, which means that it is impossible to suggest a self-consistent mathematical explanation of the preference reversal phenomenon in classical terms, which is in agreement with discussions by Tversky and Thaler (1990) and Tversky et al. (1990).

Criterion (20) not only explains the preference reversal phenomenon, but it also provides a quantitative estimate of how likely it may happen, as well as a posteriori confirmation of why it has happened. This is because the attraction factors are not just some additional arbitrary characteristics, but because their signs are prescribed by the risk aversion notion, while their values are constrained by conditions (8) – (10). Thus, due to risk aversion when facing several choices, the more certain lottery is more attractive, hence *q*(π_1_) > *q*(π_2_), which, in view of the alternation property (9), implies that *q*(π_1_) > 0, while *q*(π_2_) < 0. Contrary to this, when pricing, risk aversion is absent, hence more attractive is the lottery that can provide the larger gain, so that *q*(π_4_) > *q*(π_3_), which, again taking into account the alternation property (9), tells us that *q*(π_3_) < 0 while *q*(π_4_) > 0. Estimating the absolute values of the attraction factors by the quantity 0.25, which follows from the quarter law (10), we have the criterion

$$\begin{array}{l} -\frac{1}{2}\;<\;f\left(\pi_{2}\right)-f\left(\pi_{1}\right)\;<\frac{1}{2}\;\;. \end{array}$$

Therefore, if the given lotteries are such that their utility factors satisfy the above inequalities, we may expect that preference reversal can occur. And, vice versa, if preference reversal has happened, then the above inequalities must hold. Below we demonstrate that criterion (20) really provides a necessary and sufficient conditions for the preference reversal phenomenon.

## 4. Confirmation of preference reversal criterion

To confirm the validity of the preference reversal criterion, let us test it with empirical data of decision-making experiments. We shall consider pairs of lotteries with the notation of the previous section. The prospects, related to the choice between the lotteries *L*_1_ and *L*_2_, are denoted as π_1_ and π_2_, respectively. The prospects, corresponding to pricing of these lotteries, will be denoted by π_3_ and π_4_. The expected utility of a lottery *L* = {*x*_*i*_, *p*(*x*_*i*_)}, consisting of payoffs *x*_*i*_, with their weights *p*(*x*_*i*_), will be calculated by the formula $U\left(L\right)=\underset{i}{\sum }x_{i}p\left(x_{i}\right)$. And the utility factors are given by expression (7).

**Example 1**. Let us start with the example given by Tversky and Thaler (1990). Consider two lotteries

$$\begin{array}{l} L_{1}=\left\{4,\frac{8}{9}\;|\;0,\frac{1}{9}\right\}\;,\;L_{2}=\left\{40,\frac{1}{9}\;|\;0,\frac{8}{9}\right\}\;, \end{array}$$

whose payoffs 4 and 40 are given in some monetary units. The type of units, whether these are Dollars, or Euro, or Francs, is not of importance, since such units are canceled in definition (7) of utility factors. This is one of the advantage of employing the dimensionless utility factors that are invariant with respect to the type of payoff measures. The corresponding expected utilities

$$\begin{array}{l} U\left(L_{1}\right)=\frac{32}{9}\;,\;U\left(L_{2}\right)=\frac{40}{9}\;, \end{array}$$

result in the utility factors

$$\begin{array}{l} f\left(\pi_{1}\right)=\frac{4}{9}\;,\;f\left(\pi_{2}\right)=\frac{5}{9}\;, \end{array}$$

which show that the second lottery is more useful.

The experimental probabilities are defined as the fractions of subjects preferring the related lotteries. According to Tversky and Thaler (1990), in the case of choice, it was found that 71% of decision makers preferred the more certain lottery *L*_1_, so that

$$\begin{array}{l} p\left(\pi_{1}\right)=0.71\;>\;p\left(\pi_{2}\right)=0.29\;, \end{array}$$

despite that this lottery is less useful. In view of (4), this corresponds to the attraction factors

$$\begin{array}{l} q\left(\pi_{1}\right)=0.266\;,\;q\left(\pi_{2}\right)=-0.266\;. \end{array}$$

However, when pricing, 67% of subjects found Lottery *L*_2_ more valuable, so that

$$\begin{array}{l} p\left(\pi_{3}\right)=0.33\;<\;p\left(\pi_{4}\right)=0.67\;, \end{array}$$

despite that the win in this lottery is less probable. The related attraction factors are

$$\begin{array}{l} q\left(\pi_{3}\right)=-0.114\;,\;q\left(\pi_{4}\right)=0.114\;. \end{array}$$

Notice that, in the case of pricing, the attraction factor signs are reversed as compared to the case of choosing. This is in agreement with the probability-amount dichotomy (Kim et al., 2012): when choosing, one accepts as more attractive the lottery with a higher probability win, while when pricing, one treats as more attractive the lottery with a higher payoff amount. In the process of pricing, decision makers usually are more pragmatic, evaluating higher the more useful lottery.

Combining the data of this experiment, the two inequalities (20) read

$$\begin{array}{l} -0.228<0.111<0.452\;, \end{array}$$

which confirms the prediction of QDT.

**Example 2**. When there is no preference reversal, the criterion (20) does not hold. To illustrate this, let us consider an example treated by Tversky et al. (1990), taking the lotteries

$$\begin{array}{l} L_{1}=\left\{100,0.97\;|\;0,0.03\right\}\;,\;L_{2}=\left\{400,0.31\;|\;0,0.69\right\}\;. \end{array}$$

Their expected utilities are

$$\begin{array}{l} U\left(L_{1}\right)=97\;,\;U\left(L_{2}\right)=124\;, \end{array}$$

which yields the utility factors

$$\begin{array}{l} f\left(\pi_{1}\right)=0.439\;,\;f\left(\pi_{2}\right)=0.561\;. \end{array}$$

The first lottery is essentially more certain, and subjects overwhelmingly tend to prefer this lottery, so that

$$\begin{array}{l} p\left(\pi_{1}\right)=0.91\;>\;p\left(\pi_{2}\right)=0.09\;. \end{array}$$

According to (4), the related attractions factors are

$$\begin{array}{l} q\left(\pi_{1}\right)=0.471\;,\;q\left(\pi_{2}\right)=-0.471\;. \end{array}$$

When pricing, subjects pay higher attention to the payoff amounts so that the fraction of decision makers preferring the first lottery is drastically reduced. However, the preference reversal does not occur *per se*, with the (more narrow) majority pricing the first lottery higher:

$$\begin{array}{l} p\left(\pi_{3}\right)=0.54\;>\;p\left(\pi_{4}\right)=0.46\;. \end{array}$$

The corresponding attraction factors are

$$\begin{array}{l} q\left(\pi_{3}\right)=0.101\;,\;q\left(\pi_{4}\right)=-0.101\;. \end{array}$$

Since

$$\begin{array}{l} f\left(\pi_{2}\right)-f\left(\pi_{1}\right)=0.122<2q\left(\pi_{3}\right)=0.202\;, \end{array}$$

criterion (20) is not fulfilled, which is the expected situation in absence of preference reversal.

This example demonstrates that, although in pricing, one pays a higher attention to payoff amounts, however, the focus is not exclusively on this amount. Probabilities can also influence decisions, together with amounts.

**Example 3**. Another example from Tversky et al. (1990) deals with the lotteries

$$\begin{array}{l} L_{1}=\left\{12,0.92\;|\;0,0.08\right\}\;,\;L_{2}=\left\{175,0.06\;|\;0,0.94\right\}\;. \end{array}$$

The first lottery is both more certain as well as more useful, with the expected utilities

$$\begin{array}{l} U\left(L_{1}\right)=11.04\;,\;U\left(L_{2}\right)=10.5 \end{array}$$

and the utility factors

$$\begin{array}{l} f\left(\pi_{1}\right)=0.513\;,\;f\left(\pi_{2}\right)=0.487\;. \end{array}$$

It is not surprising that, when choosing, decision makers prefer this lottery according to

$$\begin{array}{l} p\left(\pi_{1}\right)=0.81\;>\;p\left(\pi_{2}\right)=0.19\;. \end{array}$$

The related attraction factors are

$$\begin{array}{l} q\left(\pi_{1}\right)=0.297\;,\;q\left(\pi_{2}\right)=-0.297\;. \end{array}$$

When pricing, subjects take into account that the second lottery can provide a much higher payoff, yet with too small a probability. As a result, the fraction of decision makers preferring the first lottery diminishes, but preference reversal does not happen:

$$\begin{array}{l} p\left(\pi_{3}\right)=0.58\;>\;p\left(\pi_{4}\right)=0.42\;. \end{array}$$

In pricing, the first lottery becomes less attractive than in choosing, but remains more attractive than the second lottery, with the attraction factors

$$\begin{array}{l} q\left(\pi_{3}\right)=0.067\;,\;q\left(\pi_{4}\right)=-0.067\;. \end{array}$$

In view of the relations

$$\begin{array}{l} f\left(\pi_{2}\right)-f\left(\pi_{1}\right)=-0.026\;<\;2q\left(\pi_{3}\right)=0.134\;, \end{array}$$

criterion (20) does not hold, in agreement with the absence of preference reversal. Again, we see that payoff amounts as well as probabilities are considered in the process of pricing, although the role of payoff amounts, without doubt, is more important in pricing than in choosing.

We have also analyzed a large set of data presented by Tversky et al. (1990), demonstrating the effect of preference reversal. Pairs of lotteries were presented to 198 participants. In each pair, one of the lotteries, *L*_1_, had a high probability, while the other, *L*_2_, a higher payoff with lower probability. These lotteries are given in Table 1. In each lottery, the first number is a payoff and the next number is the probability of this payoff. A lottery is represented as a set {*x, p*(*x*)}, implying that one gets either the payoff *x*, with probability *p*(*x*), or nothing, with probability 1 − *p*(*x*). The expected utilities and utility factors are shown. The first six lottery pairs include rather small payoffs. The following five pairs contain much larger payoffs by a factor of 25. And the last five pairs present a mixture of large and small payoffs. All the cases demonstrate the effect of preference reversal.

**Table 1:** **Pairs of lotteries, with their expected utilities and utility factors**.

***L*_1_**	***L*_2_**	***U*(*L*_1_)**	***U*(*L*_2_)**	***f*(π_1_)**	***f*(π_2_)**
4, 0.97	16, 0.31	3.88	4.96	0.439	0.561
2, 0.81	9, 0.19	1.62	1.71	0.486	0.514
3, 0.94	6.5, 0.50	2.82	3.25	0.465	0.535
4, 0.89	40, 0.11	3.56	4.4	0.447	0.553
2.5, 0.94	8.5, 0.39	2.35	3.315	0.415	0.585
2, 0.92	5, 0.50	1.84	2.5	0.424	0.576
50, 0.81	225, 0.19	40.5	42.75	0.486	0.514
75, 0.94	160, 0.50	70.5	80	0.468	0.532
100, 0.89	1000, 0.11	89	110	0.447	0.553
65, 0.94	210, 0.39	61.1	81.9	0.427	0.573
50, 0.92	125, 0.50	46	62.5	0.424	0.576
10, 0.78	100, 0.08	7.8	8	0.494	0.506
7, 0.69	40, 0.17	4.83	6.8	0.415	0.585
3, 0.86	13, 0.19	2.58	2.47	0.511	0.489
4, 0.94	150, 0.03	3.76	4.5	0.455	0.545
11, 0.89	135, 0.08	9.79	10.8	0.475	0.525

In Table 2, we show the prospect probabilities *p*(π_1_) and *p*(π_3_), with the corresponding attraction factors *q*(π_1_) and *q*(π_3_), demonstrating preference reversal, since *p*(π_1_) > *p*(π_2_), although *p*(π_3_) < *p*(π_4_). Those quantities that are not presented can be found from the relations

$$\begin{array}{l} f\left(\pi_{1}\right)=f\left(\pi_{3}\right)\;,\;f\left(\pi_{2}\right)=f\left(\pi_{4}\right)\;,\\ p\left(\pi_{2}\right)=1-p\left(\pi_{1}\right)\;,\;p\left(\pi_{4}\right)=1-p\left(\pi_{3}\right)\;,\\ q\left(\pi_{2}\right)=-q\left(\pi_{1}\right)\;,\;q\left(\pi_{4}\right)=-q\left(\pi_{3}\right)\;. \end{array}$$

We also show the value [*f*(π_2_)−*f*(π_1_)]/2 that has to be compared with *q*(π_3_) and *q*(π_1_) in order to check the validity of criterion (20). As is seen from Table 2, the preference reversal criterion (20) is always valid.

**Table 2:** **Probability *p*(π_1_) defined as the fraction of decision makers choosing the lottery *L*_1_, and probability *p*(π_3_) defined as the fraction of subjects pricing the lottery *L*_1_ higher**.

***p*(π_1_)**	***p*(π_3_)**	***q*(π_1_)**	***q*(π_3_)**	**[*f*(π_2_)−*f*(π_1_)]/2**
0.83	0.26	0.391	−0.179	0.061
0.68	0.22	0.194	−0.266	0.014
0.71	0.30	0.245	−0.165	0.035
0.71	0.33	0.263	−0.117	0.053
0.73	0.17	0.315	−0.245	0.085
0.62	0.14	0.196	−0.284	0.076
0.86	0.48	0.374	−0.006	0.014
0.77	0.46	0.302	−0.008	0.032
0.84	0.47	0.393	0.023	0.053
0.82	0.48	0.393	0.053	0.073
0.70	0.32	0.276	−0.104	0.076
0.81	0.38	0.316	−0.114	0.006
0.68	0.21	0.265	−0.205	0.085
0.74	0.39	0.229	−0.121	−0.011
0.74	0.38	0.285	−0.075	0.045
0.79	0.46	0.315	−0.015	0.025

*The corresponding attraction factors q*(π_1_) *and q*(π_3_), *and the combination* [*f*(π_2_)−*f*(π_1_)]/2 *that should be compared with those attraction factors according to criterion (20) obtained from QDT, which reads here q*(π_3_) < [*f*(π_2_)−*f*(π_1_)]/2 < *q*(π_1_).

Since, in each pair of lotteries considered in the case of choosing or pricing, the utility factors do not change, the preference reversal effect can be interpreted within QDT as caused by the existence of the attraction factors. If one would evaluate the lotteries solely on the basis of rational utility, no preference reversal would occur. However, preferences of decision makers involve irrational feelings and biases as well as other considerations not included in the utility, which are embodied in the attraction factors, accounting for the phenomenon of preference reversal. In order to characterize the deviation from rationality during decision making over a family of *N* trials, we can introduce the *irrationality measure*

$$\begin{array}{l} \delta_{j}\equiv \frac{1}{N}\overset{N}{\underset{n=1}{\sum }}|q\left(\pi_{j}\right)|\;. \end{array}$$

Then δ_1_ measures the level of irrationality in the course of choosing, while δ_3_ describes the degree of irrationality in the process of pricing. From Table 2, we find δ_1_ = 0.299 and δ_3_ = 0.118. Thus, people seem to be significantly more irrational when choosing, as compared to pricing. In other words, the evaluation of lotteries in pricing is more rational.

## 5. Discussion

We have shown that the phenomenon of preference reversal, which is treated as an anomaly in classical decision making, finds a natural explanation in the frame of quantum decision theory. In the latter, the preference probability consists of two terms, the utility factor quantifying the utility of a prospect, and the attraction factor characterizing behavioral biases of a decision maker. In that way, a prospect probability, defined as a quantum quantity, has the meaning of a behavioral probability taking into account both utility of the considered prospects, as well as their attractiveness for the decision maker, due to subconscious behavioral biases. We have formulated the criterion associated within QDT with preference reversal and we have illustrated its validity for a large set of empirical data.

We summarize the key steps of the logic we have followed.

1. We acknowledge the existence of risk aversion that leads human to prefer the more probable outcome ceteris paribus.
2. We formulate decisions in terms of QDT and derive the general fundamental expression (4) of QDT: *p* = *f* + *q*.
3. We interpret *q* as an “attraction factor” embodying the point resulting from risk aversion, which determines the sign of *q*.
4. The structure of QDT leads to criterion (20) for preference reversal to occur, which relates the utilities of the two lotteries to the attraction factors under choosing vs. pricing.
5. We showed that this criterion is verified by experiments.

We have thus demonstrated that QDT predicts the existence of two inequalities for the reversal to occur, that turn out to be confirmed.

It is worth noting that the effect of preference reversal does not only occur when choice is compared with pricing, but similar reversals can happen in other cases. As another illustration, we can mention the so-called planning paradox that can be represented by the following stylized example.

Suppose one is deliberating about stopping smoking. Let the imaginary plan to stop smoking be denoted as the prospect π_1_, while continuing smoking corresponds to prospect π_2_. The utility of not smoking clearly overweights that of smoking because of evident health reasons. In contrast, the negative feelings, connected with addiction, are yet too imaginary to influence the mood of the decision maker. We thus expect that the related attraction factors should be rather small, so that the decision is based mainly on rational grounds. Hence, the preference in this plan π_1_ is expressed by the inequality *p*(π_1_) > *p*(π_2_), implying that the majority of subjects would like to stop smoking.

However, when one has to choose to really stop smoking now (but not in the future), then one actually meets another alternative: really stop smoking, which can be denoted as the prospect π_3_, or continue smoking, the prospect π_4_. Deciding whether to really stop smoking now, one immediately confronts negative feelings anticipating the suffering resulting from addiction. This translates into the appearance of a negative attraction factor *q*(π_3_) devaluating the utility of not smoking. As a result, *p*(π_3_) becomes smaller than *p*(π_4_), which means that the majority of people do not really quit smoking.

This planning paradox gives a clear example of preference reversal, which cannot be understood in terms of classical utility considerations, since the utility of prospects does not change. But there is no paradox in quantum decision theory, where the effect of preference reversal is explained by the variation of attraction factors. Numerous data, collected by Walsh and Sanson-Fisher (2001) from the World Health Organization, confirm the robust existence of the preference reversal in the stop-smoking planning paradox. Thus the preference reversal is a rather general phenomenon that obtains a straightforward explanation in the framework of quantum decision theory.

### Conflict of interest statement

The authors declare that the research was conducted in the absence of any commercial or financial relationships that could be construed as a potential conflict of interest.
